# Mineral Metabolism Assays, Central DXA, and Fracture Risk Probabilities in Menopausal Patients with Non-Functional Adrenal Tumors with/Without Mild Autonomous Cortisol Secretion: Does the Presence of Unilateral Versus Bilateral Tumors Matter?

**DOI:** 10.3390/life15101639

**Published:** 2025-10-21

**Authors:** Alexandra-Ioana Trandafir, Mara Carsote, Mihai Costachescu, Oana-Claudia Sima, Alexandru-Florin Florescu

**Affiliations:** 1 PhD Doctoral School of “Carol Davila” University of Medicine and Pharmacy, 020021 Bucharest, Romania; alexandra-ioana.trandafir@drd.umfcd.ro; 2 Department of Endocrinology, “Carol Davila” University of Medicine and Pharmacy, 020021 Bucharest, Romania; 3 Department of Clinical Endocrinology V, “C.I. Parhon” National Institute of Endocrinology, 011863 Bucharest, Romania; oana-claudia.sima@drd.umfcd.ro; 4 Department of Radiology and Medical Imaging, “Dr. Carol Davila” Central Military University Emergency Hospital, 010825 Bucharest, Romania; mihai.costachescu@drd.umfcd.ro; 5 Department of Endocrinology, “Grigore T. Popa” University of Medicine and Pharmacy, 700115 Iasi, Romania; alexandru-florin.florescu@umfiasi.ro; 6 Department of Endocrinology, “Sf. Spiridon” Emergency County Clinical Hospital, 700115 Iasi, Romania

**Keywords:** osteoporosis, osteopenia, fracture, endocrine, hormone, ACTH, cortisol, CT, dexamethasone, incidentaloma

## Abstract

Introduction/Background: Most adrenal incidentalomas (AIs) are non-functioning adrenal tumors (NFATs) without clinically overt hormonal hypersecretion; one-third show subtle endocrine over-activity and mild autonomous cortisol secretion (MACS). One out of ten NFATs involves not a unilateral (UTs), but bilateral tumors (BTs). Bone health, as opposed to cardio-metabolic complications, is less studied in NFAs/MACS, particularly in BTs. Hence, we aimed to analyze (blood) mineral metabolism assays (MMAs), including bone turnover markers (BTMs), central Dual-Energy X-ray Absorptiometry (DXA), and 10-year fracture risk estimation (FRAX/FRAXplus) in menopausal patients with UTs vs. BTs. Methods: This was a retrospective, single-center study. The inclusion criteria were women aged ≥50 y and CT-based AI detection. The exclusion criteria were medication against osteoporosis, malignancies, bone metabolic disorders, and cs-1mg-DST >5 µg/dL. Results: The cohort [N = 129; mean age: 62.39 ± 7.9 y; and y since menopause (YSM): 13.7 ± 8] included UT (62.22%) and BT (31.78%) groups with a similar age, YSM, type 2 diabetes rate (35.23% vs. 36.59%), arterial hypertension (73.6% vs. 75.5%), BMI, fasting glycemia, and glycated hemoglobin A1c (*p* > 0.5 for each). The borderline significance for morning cortisol was higher in UTs vs. BTs [median (interquartile interval): 13.9 (11.16, 15.00) vs. 10.10 (8.88, 12.95) µg/dL; *p* = 0.05] and the MACS-positive rate (24.45% vs. 36.59%; *p* = 0.051). The largest tumor diameter was similar (2.26 ± 0.97 vs. 2.51 ± 0.87 cm; *p* = 0.175), as was cs-1mg-DST [1.27 (1.01, 1.95) vs. 1.52 (0.92, 2.78) µg/dL; *p* = 0.357]. MMAs, BTMs, and DXA-BMD/T scores were similar in the UT vs. BT groups. The most prevalent DXA categories were osteopenia (50.82%) and normal (41.38%). The rate of DXA bone impairment (osteoporosis + osteopenia) was 72.13% vs. 58.62%. A generally low prevalence of fragility fractures was found (3.88%; N = 5, 3/2 between the groups). Out of the 25.58% (N = 33) females who were found to be MACS-positive, 54.55% were in the UT group and 45.45% were in the BT group. Age, YSM, the rate of analyzed comorbidities, BMI, biochemical parameters, DXA/BMDs, and FRAX/FRAXplus (lumbar BMD adjustment)-based probabilities were similar between the UT and BT groups, regarding MACS-positive vs. MACS-negative groups. Diabetic patients were all MACS-positive. A higher PTH level in the MACS-positive UT vs. MACS-positive BT groups (36.32 ± 9.21 vs. 51.65 ± 9.58 pg/mL; *p* = 0.01) was found, with the mean 25-hydroxyvitamin D showing mild deficiency (24.21 ± 12.73 vs. 26.16 ± 9.89 ng/mL; *p* = 0.694). In UTs, the largest tumor diameter statistically significantly correlated with baseline ACTH (r = −0.391; *p* < 0.001) and cs-1mg-DST (r = 0.306; *p* < 0.001), while in BTs, the largest diameter of the two tumors showed a positive correlation with cs-1mg-DST (r = 0.309; *p* = 0.012). Conclusions: The findings from this real-life setting (similar age, YSM, and diabetes and MACS-positive rates) could help us to better understand the bone features in UTs vs. BTs, noting that ACTH/cs-1mg-DST measurements showed no difference. The study population was associated with a generally low fracture prevalence and 10-year fracture risk probabilities, which might act as a bias in this distinct clinical exploration. Whether a multifactorial algorithm is needed to provide a 360-degree perspective of the bone health assessment in these patients remains an open matter. So far, starting from the current guidelines, a patient-centered approach is mandatory. To our best knowledge, this study adds to the limited number of prior studies regarding bone impairment in bilateral tumors.

## 1. Introduction

Adrenal incidentalomas are associated with an elevated incidence in adults due to increasing access to thoracic, abdominal, and/or pelvic imaging evaluations, such as ultrasound, computed tomography (CT), or magnetic resonance imaging [1,2,3]. The reported prevalence varies from 0.5% to 20% in selected subgroups, the incidence being age-related (most affected individuals are aged 50 years or older) [4,5,6].

The majority of adrenal incidentalomas are non-functioning adrenal adenomas of the cortex, but approximately 20% to 40% of these tumors still show a (mild) active hormonal profile (other than overt Cushing’s syndrome) in apparently asymptomatic patients, namely, mild autonomous cortisol secretion (MACS) [7,8,9]. While variations and gaps in their hormonal characterization have been described, currently, there is a unanimous agreement to define this profile based on the second-day plasma cortisol level after screening using a 1 mg dexamethasone suppression test (DST) with a value between 1.8 and 5 µg/dL, in the absence of the typical Cushing’s syndrome-related phenotype [10,11,12]. This hormonal anomaly might represent the pathogenic mechanism causing a higher risk of cardiovascular, metabolic, and osseous complications in these subjects, noting that even non-MACS individuals have been found at higher risk, too, according to some studies, which highlights the suboptimal diagnosis criteria we have so far [13,14,15,16].

Osteoporosis and fragility fractures have been confirmed more often in certain population subgroups diagnosed with this type of tumors (±MACS-positive), especially if a multilayered panel of contributors (other than persistent hypercortisolism) is co-present, such as menopausal status, male hypogonadism, type 2 diabetes mellitus, vitamin D deficiency, etc. [12,17,18]. Of note, as opposed to many studies in the cardio-metabolic domain, bone health evaluation amid non-functioning adrenal adenomas without clinically overt hormonal hypersecretion has been less studied, and many areas are still a matter of debate.

Generally, one out of ten patients diagnosed with an adrenal tumor has bilateral tumor masses (the reported prevalence of bilateral adrenal incidentalomas varies between 7.5% and 15%), and the field of non-functioning adrenal tumors without clinically overt hormonal hypersecretion makes no exception (with some of these tumors being identified in patients that harbor various pathogenic variants of the *ARMC5* or *NR3C1* genes, etc.) [19,20,21]. A heterogeneous landscape of data has shown discordant results concerning the estimation of cortical adenoma-associated bilateral disease being prone to a higher risk of the mentioned complications versus unilateral tumors [22,23,24].

Globally, despite a guideline-based strategic workup, real-life settings have shown a suboptimal evaluation in patients with accidentally detected tumor masses at the adrenal level, and a patient-centered approach is mandatory, taking into consideration the constellation of all potential tumor-related complications and overlapping co-morbidities [25,26,27,28].

### Objective

We aimed to analyze (blood) mineral metabolism assays (including bone turnover markers), central Dual-Energy X-ray Absorptiometry (DXA), and the 10-year fracture risk estimation based on the Fracture Risk Estimator Tool (FRAX and FRAXplus) in menopausal patients who were confirmed with unilateral (UTs) versus bilateral (BTs) tumors, regardless of a positive or negative MACS profile.

## 2. Materials and Methods

### 2.1. Study Design

This was a retrospective, single-center, real-life study conducted in menopausal women (between January 2023 and January 2025, with ethical approval (7634) obtained on 4 April 2025) who were confirmed with non-functioning adrenal tumors without clinically overt hormonal hypersecretion that were accidentally detected (adrenal incidentalomas).

### 2.2. Study Population

The inclusion criteria were as follows: age of 50 years or older; menopausal status in terms of a minimum of 1 year since last menstruation (either physiological or surgical menopause); accidental confirmation of an adrenal tumor at CT scan, and then the tumor was proved to be non-functioning at endocrine assessment; available parameters upon central DXA at lumbar, total hip, and femoral neck (a time window between CT and the DXA scan of maximum 1 month). The exclusion criteria were as follows: prior/current (specific) medication against osteoporosis, active malignancies or bone metabolic disorders, prior/current hormonally active endocrine tumors (e.g., acromegaly, prolactinoma, primary hyperparathyroidism, etc.), diagnosis of neuroendocrine neoplasia at any point in life, previous confirmation of a familial endocrine syndrome, corticotherapy (current or prior 2 years), insulin therapy, unilateral or bilateral adrenalectomy, chronic kidney disease, second-day plasma cortisol after 1 mg DST above 5 µg/dL, suggestive clinical features of Cushing’s syndrome, suspected or confirmed adrenal malignancy (based on either clinical, hormonal or imaging workup).

### 2.3. Study Protocol

The demographic features included: age, years since menopause, co-diagnosis of (based on patients’ medical records) type 2 diabetes, arterial hypertension, and dyslipidemia (regardless of specific medication). Body mass index calculation (BMI) (weight/height^2^) was expressed in kg/m^2^. Abdominal CT scans were revised as second opinion by a trained radiologist (M.K.). The patients were then assigned as study group: either group UT or group BT based on CT features (unilateral tumor or bilateral tumor masses). The largest diameter was used for the analysis (including the largest diameter of the two/bilateral tumors).

Biochemical parameters were: fasting glycaemia (mg/dL), serum creatinine (mg/dL), and glycated hemoglobin A1c (%). Hormonal assays included: baseline plasma morning adrenocorticotropic hormone (A CTH) (pg/mL), and cortisol (µg/dL), as well as second-day plasma cortisol after 1 mg DST (µg/dL). MACS profile was defined as a value less than 5 µg/dL and higher or equal to 1.8 µg/dL. Mineral metabolism assays included total serum calcium (and calculated ionized calcium) in mg/dL, phosphorus (mg/dL), magnesium (mg/dL); mineral metabolism-related hormones: 25-hydroxyvitamin D (ng/mL) and parathyroid hormone (PTH) in pg/mL. Bone turnover markers included bone formation markers osteocalcin (ng/mL), P1NP (procollagen-N-terminal-peptide) in ng/mL, and total alkaline phosphatase (U/L), respectively, resorption marker serum CrossLaps (ng/mL).

**Figure 1:**
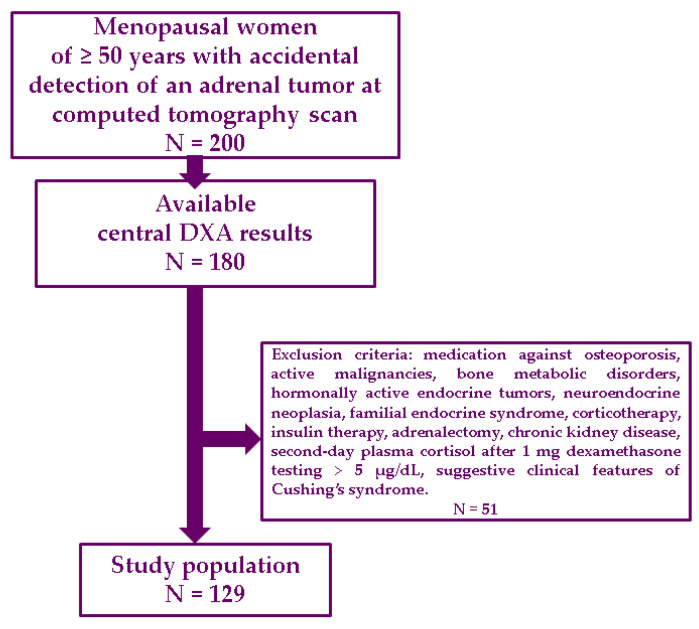
Flowchart of study protocol according to our methods.

Central DXA (GE Lunar Prodigy device) was performed at lumbar spine, total hip, and femoral neck, and provided bone mineral density (BMD) in g/sqcm, and associated T-score and Z-score. Normal DXA, osteopenia and osteoporosis were defined according to traditional cutoffs of the lowest T-score (−1 SD, respectively, −2.5 SD) [29]. FRAX was used based on the official website calculation for our country [29], and provided 10-year fracture risk for major osteoporotic fractures (MOF) without/with femoral neck BMD, and 10-year hip fracture risk (HF) without/with femoral neck BMD [30]. FRAXplus was applied upon access from (beta testing) website [31] and provided 10-year fracture risk for MOF and HF with lumbar BMD adjustment. Prevalent fragility fractures were registered based on the patients’ records and screening X-ray of the thoracic-lumbar spine (Figure 1).

### 2.4. Statistical Analysis

The distribution of continuous variables was assessed for normality using the Kolmogorov–-Smirnov test and visual inspection of histograms. Continuous variables were expressed as mean ± standard deviation (SD) for normally distributed data, or as median and quartiles (Q1, Q3) for non-normally distributed data. Categorical variables were presented as absolute numbers and percentages. Differences between categorical variables were evaluated using the Chi-square test or Fisher’s exact test, depending on cell counts. For comparisons of continuous variables between two independent groups, the independent samples *t*-test was applied when assumptions were met, and the Mann–Whitney U test was used otherwise. For comparisons across more than two groups, one-way analysis of variance (ANOVA) was employed for normally distributed variables, while the Kruskal–Wallis test was applied for non-normally distributed data. Correlation analyses were conducted using Kendall’s tau coefficient. A two-tailed *p*-value < 0.05 was considered statistically significant. No posthoc power analysis was performed, as this was a retrospective, real-life, and exploratory study. Statistical analyses were performed using SPSS v.29.0.2.0, IBM Corp., Armonk, NY, USA and using Excel v16.100.1, Microsoft, Redmond, WA, USA.

## 3. Results

A total of 129 patients were analyzed, with a mean age of 62.39 ± 7.90 years, respectively, years since menopause of 13.70 ± 58.00 and mean BMI of 29.94 ± 5.68 kg/sqm. Of these, 68.22% represented group UT, respectively, 31.78% group BT. Demographic parameters, as well as mentioned comorbidities and biochemical profile was similar between the these groups (Table 1).

Morning plasma cortisol was higher in group UT versus BT [13.9 (11.16, 15.00) versus 10.10 (8.88, 12.95) µg/dL, *p* = 0.05]. Also, a borderline significant difference showed less patients with MACS-positive profile in group UT versus BT (24.45% versus 36.59%, *p* = 0.051). The largest tumor diameter was similar between the groups of 2.26 ± 0.97 versus 2.51 ± 0.87 cm (*p* = 0.175) (Table 2).

Mineral metabolism parameters and bone turnover markers were similar between the groups UT and BT (Table 3).

The prevalence of osteoporosis, osteopenia, and normal DXA category at central DXA scan, as well as 10-year fracture risk probabilities were similar in group UT and group BT (Table 4).

**Figure 2:**
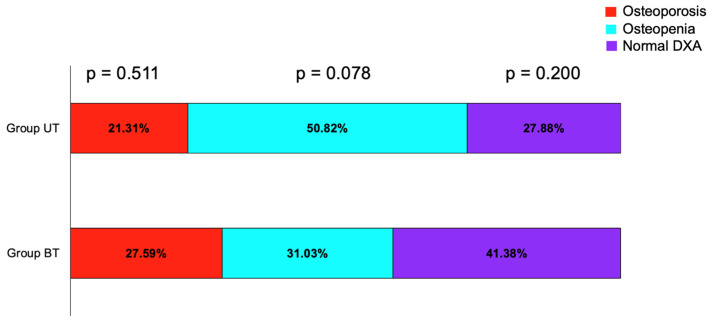
Bar chart showing percentage of patients with osteoporosis, osteopenia, and normal DXA in group UT and BT.

More than half of the women in group UT had osteopenia, while most prevalent DXA category in patients with bilateral adrenal accidentally detected masses was the normal DXA category (Figure 2).

Age -group analysis showed a similar distribution between the two studied sub-groups (*p* = 0.969) (Table 5).

**Figure 3:**
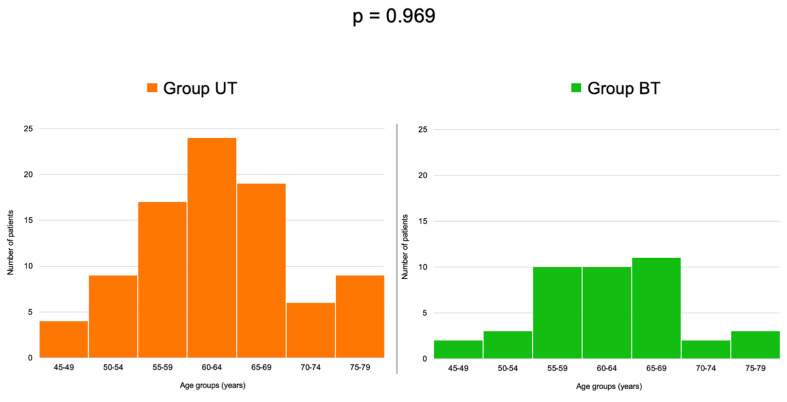
Histograms showing the distributions of patients in age-based sub-groups within group UT group and group BT group.

The highest rate of patients were in the 60–64 years group and the 65–69 years subgroup, respectively (Figure 3).

Out of the 25.58% (N = 33) females who were found MACS-positive in the entire cohort, 54.55% of patients (N = 18) were from the group UT and 45.45% from the group BT (N = 15). Age, years since menopause, the rate of analyzed comorbidities, BMI and biochemical parameters were similar between group UT and group BT, regarding the sub-category of MACS-positive and MACS-negative tumors (Table 6).

**Figure 4:**
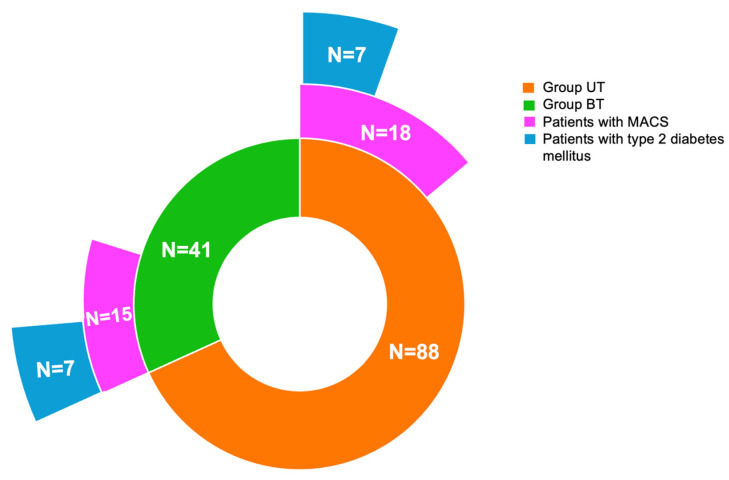
Multi-level doughnut chart showing the number of patients with MACS and type 2 diabetes from group UT and group BT.

Of note, in the UT group, 7 (46.67%) of 15 MACS patients had type 2 diabetes mellitus, whereas in the BT group, 7 (38.89%) of 18 patients had type 2 diabetes mellitus (Figure 4).

The adrenal hormonal panel and the largest tumor diameter were similar between group UT and group BT within MACS-positive and MACS-negative categories (Table 7).

**Figure A1:**
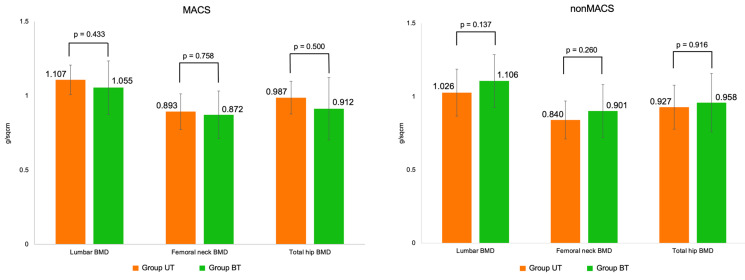
Bar chart showing mean lumbar BMD, femoral neck BMD, and total hip BMD with MACS-positive tumors (**left**) and MACS-negative profile (**right**) in UT group and BT group.

A higher PTH in MACS-positive group UT versus MACS-positive group BT (of 36.32 ± 9.21 versus 51.65 ± 9.58 pg/mL, *p* = 0.01) was found. Bone turnover markers were similar between MACS-positive patients in UT versus BT groups, as similarly found for the central DXA assessment and, FRAX- and FRAXplus-based probabilities (Table 8 and Figure A1).

**Figure 5:**
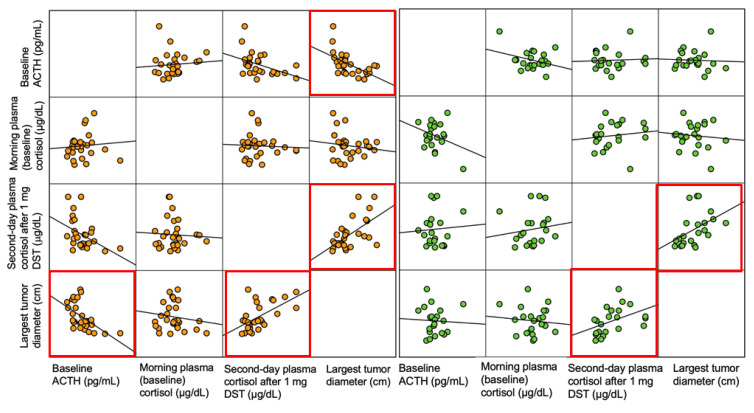
Scatterplot matrix showing the correlations regarding ACTH–-cortisol assays and the largest tumor diameter in group UT (orange) and BT (green); red box means statistically significant results.

In the group UT, the largest tumor diameter was negatively correlated with baseline ACTH (r = −0.391; *p* < 0.001) and positively correlated with second-day plasma cortisol after 1 mg DST (r = 0.306; *p* < 0.001), while in the BT group, the largest diameter of the two tumors showed a positive correlation with the second-day plasma cortisol after 1 mg DST (r = 0.309; *p* = 0.012) (Figure 5).

No statistically significant correlation was found for the adrenal hormonal parameters with BMD and 10-year fracture probabilities in either group (Table 9).

**Figure A2:**
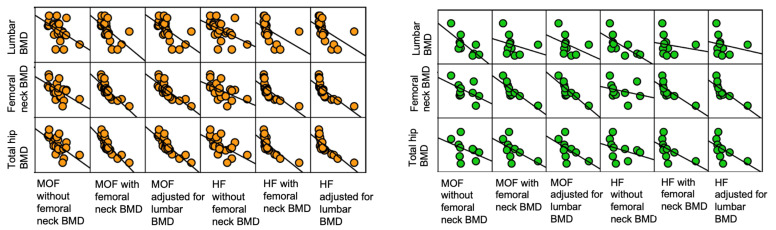
Scatterplot matrix showing the correlations (positive or negative) between DXA evaluation and 10-year fracture risk estimation in UT group (orange) and BT group (green).

In group UT, 10-year fracture risk probabilities statistically significantly correlated with lumbar, femoral neck, and total hip BMD, with correlation coefficients varying from −0.823 [between HF adjusted for lumbar BMD (*p* < 0.001) and total hip BMD] to −0.302 [between MOF without femoral neck BMD and lumbar BMD (*p* = 0.004)]. In group BT, 10-year fracture risk probabilities negatively correlated with femoral neck BMD, with correlation coefficients between −0.905 and −0.473. MOF and HF with femoral neck BMD, as well as MOF and HF adjusted for lumbar BMD negatively correlated with total hip BMD, with correlation coefficients between −0.606 and −0.514 (Table 10 and Figure A2).

## 4. Discussion

We conducted a retrospective study in consecutive menopausal females (N = 129) who were accidentally detected with adrenal tumor masses (adrenal incidentalomas) amid CT scan and, after the confirmation of a non-secretor status, they have been evaluated for the bone profile in relationship with uni/bilateral gland involvement (68.22% versus 31.78% from the original cohort). Currently, the identification of a non-functioning adrenal adenoma without clinically overt hormonal hypersecretion does not represent *per se* a well-established risk factor for low BMD and/or osteoporotic fractures, but growing evidence suggested it [32].

Notably, the highest concern involves MACS-positive sub-category (previously designated as subclinical Cushing’s syndrome [33]), and the term “MACS-related osteoporosis” [34] has been recently proposed; despite there is no specific stratified protocol, neither a general consensus of bone health evaluation in these patients [35,36,37]. Globally, numerous endocrine (either autoimmune or tumor) ailments have been reported to associate anomalies of the bone status in certain population categories without overt Cushing’s syndrome, and they are not listed as direct contributors to secondary osteoporosis (e.g., Conn’s syndrome, pheochromocytoma, etc.) [33,36,37,38]. In this cohort, 25% of the subjects displayed a MACS-positive status, slightly more often in BT (36.59%) than found in UT (25.58%), with a similar tumor size (of 2.51 versus 2.26 cm; *p* > 0.5). If MACS represents the main culprit of the bone damage, this rather similar MACS prevalence in each group might explain why the skeleton status in terms of mineral metabolism assays and DXA assessment was similar in the females with a single adenoma when compared to with those with bilateral adrenal masses (without clinically overt hormonal hypersecretion).

Subjects with a unilateral tumor and a second day plasma cortisol after DST above 1.8 µg/dL displayed osteopenia as the most common DXA category, while in MACS-positive patients with bilateral incidentalomas, one third involved each DXA category. In MACS-negative individuals, the most prevalent categories were osteopenia, and normal DXA, respectively. This overall distribution did not reach a statistical significance. For instance, Favero et al. [39] applied in a longitudinal arm of a study (N = 83) a different cut-off of the plasma cortisol after DST and found a similar osteoporosis prevalence (N = 28, 18%) in patients with a value of <1.2 µg/dL versus those with ≥1.2 µg/dL (N = 57, 35%, *p* = 0.11), thus suggesting that the cortisol cut-off after DST might not be the single discriminator of an osteoporotic status [39].

Another potential culprit in the bone health might be the age and the duration of menopause, which were similar between the study groups. Recent data suggested that the evaluation of osteoporosis/fracture risk should be gender-based in individuals identified with these adenomas without clinically overt hormonal hypersecretion. For instance, Puglisi et al. [40] analyzed a similar cohort with ours, including females [N = 120, median age of 59, interquartile interval (IQR) between 53 and 67], as well as males (N = 69, median age of 62, IQR between 56 and 68) diagnosed with non-functioning adrenal tumors without clinically overt hormonal hypersecretion. The two subgroups had a similar rate of cardio-metabolic complications, but females had a higher prevalence of bone impairment, which was defined based on DXA results (osteopenia plus osteoporosis): 76% versus 50% (*p* = 0.03). In the present study [N = 129, with a median age of 63 and an IQR between 57 and 67 years; N for the UT group = 88; median age of 63 (57 67) years; respectively, N for BT group = 41, median age of 62 (56 65) years], the rate of bone impairment was of 67.77% (72.13%, respectively, 58.62%, *p* > 0.5) [40]. On the contrary, a recent meta-analysis published in 2024 did not find that age, nor sex predicted fracture risk in relationship with the presence of these tumors [41].

Generally, this study population showed a reduced number of prevalent fractures (3.88%). The rate was similar in group UT (N = 3 fractures, representing 3.41%) versus group BT (N = 2 fractures, 4.88%), noting all of them were vertebral fractures, except for one female who had a history of a fragility fracture at the level of the third distal radius (group BT). For example, Zavatta et al. [42] found a much higher rate of fragility fractures: 24.1% in non-functioning adrenal tumors (MACS-free) (N = 474, average age of 60.1 years), respectively, 34% in MACS-positive adenomas (N = 238, average age of 65.5 years) [42]. Probably, a more refined analysis, such as vertebral morphometry, might prove to be an essential tool in the daily evaluation of these patients [43].

Another culprit might be the co-presence of type 2 diabetes mellitus, which generally has been found with a higher prevalence than age- and sex-matched (tumor-free) controls [44,45]. In this instance, we identified a rate of 35.23% in group UT which was not statistically significant different from the one in group BT (of 36.59%). The prior mentioned study of Favero et al. [39] showed a lower rate of type 2 diabetes of 7.1% (N =2/28) versus 24.6% (N = 14/57) in patients below and above the cutoff of 1.2 µg/dL for the second day plasma cortisol after DST [39]. Whether obesity is an additional contributor remains an open issue. A study in 49 patients with MACS and non-MACS adenomas found a positive correlation between plasma cortisol following DST and the subcutaneous fat area (r = 0.3; *p* = 0.048). The muscle–-fat–-bone–-adrenal cross-talk might play a role in this specific instance [46].

Blood assays of the mineral metabolism revealed a similar profile between the groups UT and BT, as well as the MACS sub-analysis. There was only one exception: PTH in MACS-positive-BT versus UT was higher, but with intra-normal variations, noting that 25-hydroxyvitamin D and serum calcium levels were not different in order to suspect a component of secondary hyperparathyroidism. The mean serum vitamin D levels revealed a mild deficiency in both groups. The bone turnover markers pointed out a similar profile, with average values within normal limits. The prior mentioned study of Zavatta et al. [42] also detected similar values of beta-CTX (0.352 versus 0.354 ng/mL, *p* = 0.884) between MACS-negative and MACS-positive adenomas [42].

In addition, DXA-BMD pinpointed similar values between patients with a unilateral versus bilateral tumors. This might be explained by the homogenous panel of potential contributors, as mentioned (age, years since menopause, respectively, diabetes and MACS prevalence) in the two groups. While ACTH/cortisol assays showed statistically significant correlations with the largest tumor size, we found no association with BMD/T-score at DXA. Of note, similar ACTH levels were found in MACS in comparison to non-MACS. It is interesting considering that a mild cortisol secretion could have a slight effect in lowering ACTH levels. A supplementary explanation might be a study population with a relative low fracture risk, as reflected by mean T-scores within normal DXA category in UT and BT groups or low FRAX-derive probabilities (the highest values being of 5.1%, respectively, 3.05% for MOF, respectively, and HF). We mention, for example, an analysis conducted by Han et al. [47] in a group of MACS-positive (N = 11) versus MACS-negative (N = 34) patients, and no statistically significant correlation between lumbar BMD and 24-h free urinary cortisol was found [47]. Moraes et al. [48] found a single correlation in a cohort of 85 patients (45 subjects with non-MACS and 30 with MACS-positive tumors) with concern to the second day plasma cortisol after DST and bone measurements, specifically, Meta/Inn.vBMD ratio at high-resolution peripheral quantitative CT (HR-pQCT) (r = 0.29; *p* = 0.01) [48]. Some studies suggested another type of correlation between a hormonal assay and DXA-based parameters in terms of using the levels of serum androgens, not cortisol (which may be found low in certain sub-categories with adrenal incidentalomas via a low ACTH). But routine use of androgens assays as cutoff diagnostics criteria or risk factors for an abnormal bone status is not currently accepted [49].

In the group UT, the 10-year fracture risk probabilities statistically significantly correlated with lumbar, femoral neck, and total hip BMD, with correlation coefficients varying from −0.823 [between HF adjusted for lumbar BMD (*p* < 0.001) and total hip BMD] to −0.302 [between MOF without femoral neck BMD and lumbar BMD (*p* = 0.004)]. In the group BT, 10-year fracture risk probabilities negatively correlated with femoral neck BMD, with correlation coefficients between −0.905 and −0.473. MOF and HF with femoral neck BMD, as well as MOF and HF adjusted for lumbar BMD correlated with total hip BMD, with correlation coefficients between −0.606 and −0.514. These findings are in agreement with the estimation within the general population [50].

Regarding the cardio-metabolic complications, we found similar rates of arterial hypertension (72.70% versus 75.60%), dyslipidemia of any type (79% versus 73.30%; *p* > 0.5 for each), and type 2 diabetes, as specified, including similar glycated hemoglobin A1c, in UT versus BT subjects. In this specific matter, there is no unanimous agreement, and some studies, such as ours, showed a similar rate of co-morbidities. Even more complicated panels of assays might reveal the same features in subjects diagnosed with uni versus bilateral tumors. For instance, one study in 298 consecutive individuals with adrenal incidentalomas (a rate of uni/bilateral of 24.8% versus 72.2%) pointed out a higher prevalence of abnormal endocrine profile in patients with bilateral masses (35.1% versus 17.9%; *p* = 0.003), but in this study, the “subclinical Cushing’s syndrome” was designated for a post-DST cortisol at least 1.8 µg/dL plus at least one other abnormal hormonal assay. Also, DST applied 2 mg dexamethasone for 24-h test for two days, not 1 mg overnight, which is the current norm, as applied in the present study. Yet, the results showed a similar age, BMI, tumor diameter, as well as prevalence of hypertension, type 2 diabetes and dyslipidemia in individuals with a single adenoma versus bilateral adrenal masses without clinically overt hormonal hypersecretion (no osseous evaluation was performed) [51].

A literature search highlighted only a small number of studies to specifically address the bone perspective with respect to the unilateral versus bilateral tumor involvement [52,53]. One study in 175/38 subjects with unilateral/bilateral adrenal incidentalomas showed a similar age, BMI, and cortisol secretion profile, as well as rate of high blood pressure, type 2 diabetes, and dyslipidemia, but femoral neck BMD was lower (−0.45 ± 0.86 versus 0.09 ± 1.07 g/sqcm; *p* = 0.004), and the prevalence of vertebral fractures was higher (52.60% versus 28.00%; *p* = 0.007) in adults with bilateral versus unilateral tumors [52]. Another cohort of 105/47 patients with uni/bilateral adrenal incidentalomas revealed a similar age, BMI, prevalence of diabetes, hypertension, dyslipidemia, as well as a similar DXA-based femoral neck BMD, but the rate of osteoporosis diagnosis was statistically significantly elevated in people with bilateral versus unilateral incidentalomas (31.10% versus 15.90%; *p* = 0.011) [53].

As limits of the current study, we mention the sample size and the retrospective design, noting this is a real-life setting. Yet, many of the adrenal incidentalomas nowadays might remain unexplored from the biochemical and hormonal perspective, not to mention the mineral and bone assessments. For instance, a study from 2025, published by O’Connor et al. [54] showed a guideline-based biochemical work-up was performed only in 11% of the 245 included patients who were newly diagnosed with adrenal incidentalomas [54]. Moreover, as opposite to an extensive evaluation of the cardio-metabolic complications in non-functioning adrenal tumors ± MACS, the fracture risk and osseous status is less explored, and even less with respect to uni/bilateral involvement [52,55]. Nevertheless, looking for osteoporosis in menopausal subgroup seems essential to provide a 360-degree perspective of the adrenal tumor-associated clinical landscape. Moreover, we did not include the patients who underwent adrenalectomy, which might positively impact the bone features according to post-surgery longitudinal observations, as opposite to a conservative management [56,57]. While we excluded patients who were offered specific anti-osteoporotic regimes, we did not quantify the vitamin D and calcium supplementation since we analyzed the blood mineral metabolism assays (serum calcium and 25-hydroxyvitamin D) as biomarker surrogates. Neither had we enough data to analyze the potential impact of anti-diabetic drugs on bone health. Also, a trabecular bone score (TBS) analysis might help navigating the assessment of microarchitecture damage, but TBS was available only for a small sub-group, with no consistent records. Moreover, further expansion to pre-menopausal women and males might help the understanding of the bone–-adrenal interplay in this specific matter, as well as a longitudinal study to observe the incidental fractures.

In addition to a mandatory awareness of the bone profile in these patients, further research might include multifactorial algorithms to pinpoint the high-risk subcategory and suggest an interventional strategy with a double target: adrenal tumor and bone health (e.g., adrenalectomy or initiating anti-osteoporotic drugs at higher DXA-BMD or lower FRAX thresholds than applied in the general population).

## 5. Conclusions

The findings of this real-life setting (similar age, years since menopause, and diabetes and MACS-positive rates) might explain the bone features in the UT group versus the BT group, noting that ACTH/cortisol post-DST measurements showed no difference. The study population was associated with generally low fragility fracture prevalence and 10-year fracture risk probabilities, which might act as a bias in this distinct clinical exploration. Whether a multifactorial algorithm is needed to provide a 360-degree perspective of the bone health assessment in these patients remains an open matter. So far, starting from the current guidelines, a patient-centered approach is mandatory. To our best knowledge, this study adds to the limited number of prior studies regarding bone impairment in bilateral versus unilateral adrenal tumors/incidentalomas.

## Figures and Tables

**Table 1:** Demographic characteristics and biochemical profile of the entire cohort, group UT and group BT (abbreviations: BT = bilateral tumors; BMI = body mass index; N = number of patients; SD = standard deviation; UT = unilateral tumor).

Parameter	Entire Cohort (N = 129, 100%)	UT Group (N = 88, 68.22%)	BT Group (N = 41, 31.78%)	*p*-Value	Normal Range
Age (years), mean ± SD	62.39 ± 7.90	62.72 ± 7.88	61.68 ± 8.00	0.492	
Years since menopause, mean ± SD	13.70 ± 8.00	13.62 ± 8.47	13.91 ± 7.15	0.887	
Type 2 diabetes mellitus, N (%)	46 (35.66)	31 (35.23)	15 (36.59)	0.881	
Arterial hypertension, N (%)	95 (73.60)	64 (72.70)	31 (75.60)	0.729	
Dyslipidemia, N (%)	71 (77.20)	49 (79.00)	22 (73.30)	0.542	
BMI (kg/sqm), mean ± SD	29.94 ± 5.68	29.80 ± 5.00	30.24 ± 7.05	0.706	
Fasting glycaemia (mg/dL), mean ± SD	106.68 ± 27.85	107.59 ± 31.69	105.14 ± 20.30	0.741	74–106
Glycated hemoglobin A1c (%), mean ± SD	6.12 ± 1.16	6.17 ± 1.39	6.03 ± 0.58	0.656	4.8–5.9
Serum creatinine (mg/dL), mean ± SD	0.77 ± 0.18	0.75 ± 0.18	0.80 ± 0.18	0.366	0.7–1.2

**Table 2:** Hormonal (adrenal) profile of the entire cohort, group UT and group BT (Abbreviations: ACTH = adrenocorticotropic hormone; BT = bilateral tumors; DST = dexamethasone suppression test; Q = quartile; MACS = mild autonomous cortisol secretion; N = number of patients; UT = unilateral tumor).

Parameter	Entire Cohort (N = 129, 100%)	UT Group (N = 88, 68.22%)	BT Group (N = 41, 31.78%)	*p*-Value	Normal Range
Baseline ACTH (pg/mL), median (Q1, Q3)	11.78 (7.89, 15.87)	12.23 (7.78, 16.6)	10.81 (8.57,15.32)	0.532	3–66
Morning plasma (baseline) cortisol (µg/dL), median (Q1, Q3)	12.97 (9.70, 14.87)	13.9 (11.16, 15.00)	10.10 (8.88, 12.95)	0.050	6.2–19.4
Second-day plasma cortisol after 1 mg DST (µg/dL), median (Q1, Q3)	1.33 (0.96, 2.21)	1.27 (1.01, 1.95)	1.52 (0.92, 2.78)	0.357	<1.8
MACS, N (%)	33 (25.58)	18 (25.45)	15 (36.59)	0.051	
Largest tumor diameter (cm), mean ± SD	2.34 ± 0.94	2.26 ± 0.97	2.51 ± 0.87	0.175	

**Table 3:** Mineral metabolism and bone turnover markers of the entire cohort, group UT and group BT (abbreviations: BT = bilateral tumors; PTH = parathyroid hormone; P1NP = procollagen-N-terminal-peptide; N = number of patients; SD = standard deviation; UT = unilateral tumor).

Parameter	Entire Cohort (N = 129, 100%)	UT Group (N = 88, 68.22%)	BT Group (N = 41, 31.78%)	*p*-Value	Normal Range
Total serum calcium (mg/dL), mean ± SD	9.55 ± 0.47	9.6 ± 0.50	9.47 ± 0.40	0.236	8.4–10.2
Serum ionized calcium (mg/dL), mean ± SD	4.12 ± 0.27	4.11 ± 0.30	4.14 ± 0.21	0.664	3.9–4.9
Serum phosphorus (mg/dL), mean ± SD	3.70 ± 0.54	3.69 ± 0.55	3.73 ± 0.54	0.738	2.5–4.5
Serum magnesium (mg/dL), mean ± SD	1.95 ± 0.22	1.94 ± 0.23	1.96 ± 0.18	0.678	1.6–2.6
25-hydroxyvitamin D (ng/mL), mean ± SD	23.76 ± 11.08	22.6 ± 10.71	26.05 ± 11.66	0.207	30–100
PTH (pg/mL), mean ± SD	45.17 ± 16.14	43.6 ± 16.29	48.06 ± 15.84	0.324	15–65
Osteocalcin (ng/mL), mean ± SD	22.98 ± 9.04	23.05 ± 11.25	24.76 ± 13.53	0.601	14–46
Alkaline phosphatase (U/L), mean ± SD	78.92 ± 28.14	79.41 ± 27.95	78.43 ± 36.47	0.893	35–129
P1NP (ng/mL), mean ± SD	56.41 ± 21.19	56.12 ± 27.03	53.41 ± 14.74	0.670	20.25–76.31
CrossLaps (ng/mL), mean ± SD	0.46 ± 0.21	0.47 ± 0.24	0.50 ± 0.28	0.596	0.33–0.782

**Table 4:** DXA assessment and FRAX-based estimation in group UT versus group BT (abbreviations: BMD = bone mineral density; BT = bilateral tumors; DXA = Dual-Energy X-ray Absorptiometry; HF = 10-year hip fracture risk; MOF = 10-year major osteoporotic fractures risk; N = number of patients; Q = quartile; SD = standard deviation; UT = unilateral tumor).

Parameter	Entire Cohort (N = 129, 100%)	UT Group (N = 88, 68.22%)	BT Group (N = 41, 31.78%)	*p*-Value	Normal Range
Osteoporosis, N (%)	21 (23.33)	13 (21.31)	8 (27.59)	0.511	
Osteopenia, N (%)	40 (44.44)	31 (50.82)	9 (31.03)	0.078	
Normal DXA, N (%)	29 (32.22)	17 (27.88)	12 (41.38)	0.200	
Prevalent fragility fractures, N (%)	5 (3.88)	3 (3.41)	2 (4.88)	0.717	
Lumbar BMD (g/sqcm), mean ± SD	1.069 ± 0.16	1.047 ± 0.15	1.090 ± 0.18	0.317	
Lumbar T-score (SD), mean ± SD	−1.08 ± 1.42	−1.20 ± 1.36	−0.82 ± 1.53	0.251	>−1
Lumbar Z-score (SD), mean ± SD	−0.15 ± 0.80	−0.13 ± 0.78	−0.21 ± 0.89	0.097	
Femoral neck BMD (g/sqcm), mean ± SD	0.865 ± 0.14	0.857 ± 0.13	0.890 ± 0.17	0.421	
Femoral neck T-score (SD), mean ± SD	−1.07 ± 0.98	−1.14 ± 0.87	−0.88 ± 1.25	0.373	>−1
Femoral neck Z-score (SD), mean ± SD	0.24 ± 1.02	0.20 ± 1.03	0.33 ± 1.04	0.710	
Total hip BMD (g/sqcm), mean ± SD	0.946 ± 0.16	0.947 ± 0.14	0.945 ± 0.20	0.974	
Total hip T-score (SD), mean ± SD	−0.62 ± 1.2	−0.57 ± 1.21	−0.7 ± 1.21	0.670	>−1
Total hip Z-score (SD), mean ± SD	0.24 ± 1.02	0.2 ± 1.03	0.33 ± 1.04	0.623	
MOF without femoral neck BMD (%), median (Q1, Q3)	5.10 (3.25, 7.25)	4.50 (3.20, 6.67)	5.20 (3.60, 7.55)	0.344	
HF without femoral neck BMD (%), median (Q1, Q3)	0.90 (0.40, 1.67)	0.90 (0.40, 1.70)	1.10 (0.45, 1.70)	0.951	
MOF with femoral neck BMD (%), median (Q1, Q3)	4.70 (3.52, 7.62)	4.80 (3.50, 7.85)	4.30 (3.60, 5.87)	0.606	
HF with femoral neck BMD (%), median (Q1, Q3)	0.70 (0.30, 1.47)	0.85 (0.30, 1.85)	0.60 (0.10, 1.05)	0.176	
MOF adjusted for lumbar BMD (%), median (Q1, Q3)	2.90 (2.17, 3.97)	3.05 (2.22, 4.12)	2.35 (2.07, 3.85)	0.381	
HF adjusted for lumbar BMD (%), median (Q1, Q3)	0.40 (0.20, 1.02)	0.45 (0.20, 1.07)	0.37 (0.10, 0.85)	0.416	

**Table 5:** Five-year age groups in the study patients.

Age Group (years)	UT Group (N = 88, 68.22%))	BT Group (N = 41, 31.78%)	*p*-Value
45–49	4 (4.55)	2 (4.88)	0.969
50–54	9 (10.23)	3 (7.32)	
55–59	17 (19.32)	10 (24.39)	
60–64	24 (27.27)	10 (24.39)	
65–69	19 (21.59)	11 (26.83)	
70–74	6 (6.82)	2 (4.88)	
70–79	9 (10.23)	3 (7.32)	

**Table 6:** Demographic characteristics and biochemical profile amid MACS confirmation in group UT versus BT (abbreviations: BT = bilateral tumors; BMI = body mass index; MACS = mild autonomous cortisol secretion; N = number of patients; SD = standard deviation; UT = unilateral tumor).

	MACS (N = 33)			Non-MACS (N = 96)		
Parameter	UT Group (N = 18, 54.55%)	BT Group (N = 15, 45.45%)	*p*-Value	UT Group (N = 70, 72.92%)	BT Group (N = 26, 27.08%)	*p*-Value
Age (years), mean ± SD	61.17 ± 9.11	61.73 ± 4.46	0.818	63.11 ± 7.56	61.65 ± 9.56	0.437
Years since menopause, mean ± SD	12.90 ± 9.56	12.38 ± 4.77	0.882	13.79 ± 8.31	14.79 ± 8.24	0.698
Type 2 diabetes mellitus, N (%)	7 (38.89)	7 (46.67)	0.653	24 (34.29)	8 (30.77)	0.745
Arterial hypertension, N (%)	13 (72.2)	12 (80)	0.604	51 (72.9)	19 (73.1)	0.983
Dyslipidemia, N (%)	13 (86.7)	10 (83.3)	0.809	36 (76.6)	12 (66.7)	0.415
BMI (kg/sqm), mean ± SD	29.70 ± 3.93	31.80 ± 6.18	0.319	29.82 ± 5.21	29.40 ± 7.48	0.773
Fasting glycaemia (mg/dL), mean ± SD	120 ± 39.98	105.11 ± 18.22	0.375	103.30 ± 27.83	105.15 ± 21.72	0.820
Glycated hemoglobin A1c (%), mean ± SD	6.28 ± 1.66	6.34 ± 0.33	0.931	6.10 ± 1.23	5.84 ± 0.63	0.482
Serum creatinine (mg/dL), mean ± SD	0.86 ± 0.24	0.78 ± 0.18	0.418	0.72 ± 0.14	0.81 ± 0.18	0.067

**Table 7:** Hormonal and CT adrenal profile in study patients with/without MACS profile (abbreviations: ACTH = adrenocorticotropic hormone; BT = bilateral tumors; MACS = mild autonomous cortisol secretion; N = number of patients; Q = quartile; UT = unilateral tumor).

	MACS (N = 33)			Non-MACS (N = 96)		
Parameter	UT Group (N = 18, 54.55%)	BT Group (N = 15, 45.45%)	*p*-Value	UT Group (N = 70, 72.92%)	BT Group (N = 26, 27.08%)	*p*-Value
Baseline ACTH (pg/mL), median (Q1, Q3)	10.47 (7.23, 16.28)	9.46 (7.56, 11.88)	0.643	12.30 (7.86, 17.48)	11.27 (8.67, 15.97)	0.937
Morning plasma (baseline) cortisol (μg/dL), median (Q1, Q3)	12.75 (10.96, 14, 49)	10.54 (7.98, 12.73)	0.057	13.03 (9.54, 14.48)	10.00 (8.50, 11.96	0.214
Second-day plasma cortisol after 1 mg DST (μg/dL), median (Q1, Q3)	2.37 (1.92, 3.75)	2.79 (2.30, 4.58)	0.395	1.05 (0.76, 1.22)	0.96 (0.88, 1.34	0.431
Largest tumor diameter (cm), mean ± SD	2.78 ± 0.94	3.10 ± 0.66	0.286	2.13 ± 0.94	2.14 ± 0.78	0.957

**Table 8:** Mineral metabolism assays, bone turnover markers, DXA, and FRAX estimation in group UT and group BT: the sub-analysis of MACS-positive and MACS-negative patients (abbreviations: BT = bilateral tumors; BMD = bone mineral density; DXA = Dual-Energy X-ray Absorptiometry; HF = 10-year hip fracture risk; MACS = mild autonomous cortisol secretion; MOF = 10-year major osteoporotic fractures risk; N = number of patients; PTH = parathyroid hormone; P1NP = procollagen-N-terminal-peptide; SD = standard deviation; UT = unilateral tumor).

	MACS (N = 33)			Non-MACS (N = 96)		
Parameter	UT Group (N = 18, 54.55%)	BT Group (N = 15, 45.45%)	*p*-Value	UT Group (N = 70, 72.92%)	BT Group (N = 26, 27.08%)	*p*-Value
Total serum calcium (mg/dL), mean ± SD	9.60 ± 0.44	9.43 ± 0.35	0.293	9.59 ± 0.53	9.49 ± 0.43	0.479
Serum ionized calcium (mg/dL), mean ± SD	4.10 ± 0.23	4.18 ± 0.20	0.391	4.11 ± 0.32	4.10 ± 0.22	0.913
Serum phosphorus (mg/dL), mean ± SD	3.63 ± 0.39	3.84 ± 0.67	0.331	3.71 ± 0.60	3.66 ± 0.46	0.789
Serum magnesium (mg/dL), mean ± SD	1.88 ± 0.29	1.91 ± 0.21	0.794	1.96 ± 0.21	2.00 ± 0.15	0.548
25-hydroxyvitamin D (ng/mL), mean ± SD	24.21 ± 12.73	26.16 ± 9.89	0.694	22.01 ± 10.01	25.97 ± 13.05	0.246
PTH (pg/mL), mean ± SD	36.32 ± 9.21	51.65 ± 9.58	0.010	46.30 ± 17.61	46.86 ± 17.55	0.921
Osteocalcin (ng/mL), mean ± SD	22.67 ± 12.60	24.83 ± 11.57	0.719	23.18 ± 10.96	24.71 ± 14.94	0.704
Alkaline phosphatase (U/L), mean ± SD	71.29 ± 25.11	83.73 ± 23.57	0.219	82.25 ± 28.63 ±	75.01 ± 43.20	0.459
P1NP (ng/mL), mean ± SD	47.61 ± 28.08	61.06 ± 22.37	0.379	60.37 ± 26.16	49.16 ± 6.80	0.087
CrossLaps (ng/mL), mean ± SD	0.46 ± 0.25	0.53 ± 0.32	0.638	0.47 ± 0.23	0.49 ± 0.27	0.784
Lumbar BMD (g/sqcm), mean ± SD	1.107 ± 0.10	1.055 ± 0.18	0.433	1.026 ± 0.16	1.106 ± 0.18	0.137
Lumbar T-score (SD), mean ± SD	−0.86 ± 1.31	−0.86 ± 1.59	0.991	−1.32 ± 1.36	−0.80 ± 1.55	0.211
Lumbar Z-score (SD), mean ± SD	−0.21 ± 1.04	−0.02 ± 1.07	0.667	−0.34 ± 1.26	0.30 ± 1.49	0.100
Femoral neck BMD (g/sqcm), mean ± SD	0.893 ± 0.12	0.872 ± 0.16	0.758	0.840 ± 0.13	0.901 ± 0.18	0.260
Femoral neck T-score (SD), mean ± SD	−0.92 ± 0.84	−0.76 ± 1.43	0.754	−1.24 ± 0.88	−0.96 ± 1.21	0.433
Femoral neck Z-score (SD), mean ± SD	0.00 ± 0.76	−0.21 ± 0.80	0.574	−0.18 ± 0.80	−0.22 ± 0.97	0.916
Total hip BMD (g/sqcm), mean ± SD	0.987 ± 0.11	0.912 ± 0.21	0.500	0.927 ± 0.15	0.958 ± 0.20	0.604
Total hip T-score (SD), mean ± SD	−0.15 ± 0.85	0.82 ± 1.25	0.165	−0.75 ± 1.31	−0.63 ± 1.22	0.787
Total hip Z-score (SD), mean ± SD	0.53 ± 0.63	0.26 ± 1.11	0.547	0.05 ± 1.14	0.37 ± 1.04	0.378
Osteoporosis, N (%)	2 (13.33)	4 (33.33)	0.357	11 (23.91)	4 (23.53)	0.975
Osteopenia, N (%)	7 (46.67)	4 (33.33)	0.484	24 (52.17)	5 (29.41)	0.108
Normal DXA, N (%)	6 (40.00)	4 (33.33)	0.722	11 (23.91)	8 (47.06)	0.076
MOF without femoral neck BMD (%), median (Q1, Q3)	3.6 (3.2, 4.5)	3.30 (2.95, 4.62)	0.496	4.60 (3.15, 7.77)	5.30 (3.50, 7.75)	0.289
HF without femoral neck BMD (%), median (Q1, Q3)	0.60 (0.30, 0.90)	0.40 (0.22,0.50)	0.970	1.10 (0.40, 2.00)	1.35 (0.37, 1.35)	0.523
MOF with femoral neck BMD (%), median (Q1, Q3)	4.10 (3.10, 4.80)	4.30 (3.85, 4.67)	0.758	5.05 (3.57, 8.62)	4.50 (3.22, 8.55)	0.723
HF with femoral neck BMD (%), median (Q1, Q3)	0.50 (0.20, 0.80)	0.40 (0.12, 0.90)	0.439	1.05 (0.45, 2.07)	0.65 (0.10, 1.35)	0.303
MOF adjusted for lumbar BMD (%), median (Q1, Q3)	2.40 (2.10, 3.10)	2.40 (2.12, 3.27)	0.642	3.45 (2.80, 4.42)	2.35 (1.92, 5.02)	0.338
HF adjusted for lumbar BMD (%), median (Q1, Q3)	0.35 (0.20, 0.80)	0.40 (0.10, 0.70)	0.876	0.60 (0.27, 1.45)	0.37 (0.10, 1.32)	0.346

**Table 9:** Correlations of adrenal (hormonal and CT) parameters with BMD and 10-year fracture risk probabilities within group UT and BT (abbreviations: ACTH = adrenocorticotropic hormone; BT = bilateral tumors; BMD = bone mineral density; DST = dexamethasone suppression test; HF = 10-year hip fracture risk; MOF = 10-year major osteoporotic fractures risk; UT = unilateral tumor).

Parameter	Baseline ACTH (pg/mL)	Morning Plasma (Baseline) Cortisol (μg/dL)	Second-Day Plasma Cortisol After 1 mg DST (μg/dL)	Largest Tumor Diameter (cm)	Lumbar BMD (g/sqcm)	Femoral Neck BMD (g/sqcm)	Total Hip BMD (g/sqcm)	MOF Without Femoral Neck BMD	MOF with Femoral Neck BMD	MOF Adjusted for Lumbar BMD	HF Without Femoral Neck BMD	HF with Femoral Neck BMD	HF Adjusted for Lumbar BMD
UT Group													
Baseline ACTH (pg/mL)		*p* = 0.237 r = 0.118	*p* = 0.089 r = −0.215	*p* < 0.001 r = −0.391	*p* = 0.925 r = 0.010	*p* = 0.407 r = −0.094	*p* = 0.154 r = −0.187	*p* = 0.406 r = −0.094	*p* = 0.605 r = 0.067	*p* = 0.807 r = 0.032	*p* = 0.535 r = −0.072	*p* = 0.707 r = 0.049	*p* = 0.749 r = 0.043
Morning plasma (baseline) cortisol (μg/dL)	*p* = 0.237 r = 0.118		*p* = 0.973 r = −0.004	*p* = 0.854 r = −0.019	*p* = 0.318 r = 0.103	*p* = 0.435 r = −0.086	*p* = 0.072 r = −0.232	*p* = 0.106 r = 0.177	*p* = 0.172 r = 0.168	*p* = 0.158 r = 0.177	*p* = 0.106 r = 0.181	*p* = 0.889 r = 0.017	*p* = 0.504 r = 0.085
Second-day plasma cortisol after 1 mg DST (μg/dL)	*p* = 0.089 r = −0.215	*p* = 0.973 r = −0.004		*p* < 0.001 r = 0.306	*p* = 0.320 r = 0.131	*p* = 0.655 r = 0.065	*p* = 0.309 r = 0.149	*p* = 0.591 r = −0.077	*p* = 0.224 r = −0.213	*p* = 0.107 r = −0.290	*p* = 0.690 r = −0.058	*p* = 0.401 r = 0.148	*p* = 0.455 r = −0.136
Largest tumor diameter (cm)	*p* < 0.001 r = −0.391	*p* = 0.854 r = −0.019	*p* < 0.001 r = 0.306		*p* = 0.908 r = 0.012	*p* = 0.844 r = 0.016	*p* = 0.358 r = 0.117	*p* = 0.341 r = 0.101	*p* = 0.474 r = 0.090	*p* = 0.354 r = 0.117	*p* = 0.427 r = 0.086	*p* = 0.504 r = 0.085	*p* = 0.535 r = 0.079
BT Group													
Baseline ACTH (pg/mL)		*p* = 0.580 r = 0.074	*p* = 0.428 r = −0.119	*p* = 0.392 r = −0.117	*p* = 0.904 r = 0.019	*p* = 0.520 r = 0.124	*p* = 0.161 r = 0.250	*p* = 0.786 r = −0.043	*p* = 0.412 r = −0.165	*p* = 0.427 r = − 0.168	*p* = 0.734 r = −0.053	*p* = 0.620 r = −0.101	*p* = 0.756 r = −0.067
Morning plasma (baseline) cortisol (μg/dL)	*p* = 0.580 r = 0.074		*p* = 0.597 r = 0.079	*p* = 0.573 r = 0.077	*p* = 0.334 r = 0.152	*p* = 0.347 r = 0.181	*p* = 0.365 r = −0.162	*p* = 0.417 r = −0.129	*p* = 0.352 r = −0.187	*p* = 0.199 r = − 0.271	*p* = 0.610 r = −0.079	*p* = 0.783 r = 0.056	*p* = 0.494 r = −0.148
Second-day plasma cortisol after 1 mg DST (μg/dL)	*p* = 0.428 r = −0.119	*p* = 0.597 r = 0.079		*p* = 0.012 r = 0.309	*p* > 0.999 r = 0.000	*p* = 0.714 r = −0.077	*p* = 0.418 r = −0.150	*p* > 0.999 r = 0.000	*p* = 0.583 r = 0.121	*p* > 0.999 r = 0.000	*p* = 0.819 r = −0.040	*p* = 0.679 r = 0.092	*p* = 0.937 r = 0.019
Largest tumor diameter (cm)	*p* = 0.392 r = −0.117	*p* = 0.573 r = 0.077	*p* = 0.012 r = 0.309		*p* = 0.432 r = −0.124	*p* = 0.692 r = −0.077	*p* = 0.680 r = 0.174	*p* = 0.904 r = 0.019	*p* = 0.742 r = 0.066	*p* = 0.760 r = −0.065	*p* = 0.820 r = 0.036	*p* = 0.912 r = 0.023	*p* = 0.803 r = −0.054

**Table 10:** Correlations between BMD and 10-year fracture risk probabilities (abbreviations: BT = bilateral tumors; BMD = bone mineral density; HF = hip fracture; MOF = major osteoporotic fracture; UT = unilateral tumor).

Parameter	MOF Without Femoral Neck BMD	MOF with Femoral Neck BMD	MOF Adjusted for Lumbar BMD	HF Without Femoral Neck BMD	HF with Femoral Neck BMD	HF Adjusted for Lumbar BMD
**UT Group**						
Lumbar BMD (g/sqcm)	*p* = 0.004 r = −0.302	*p* < 0.001 r = −0.419	*p* < 0.001 r = −0.406	*p* = 0.004 r = −0.309	*p* < 0.001 r = −0.418	*p* < 0.001 r = −0.419
Femoral neck BMD (g/sqcm)	*p* < 0.001 r = −0.388	*p* < 0.001 r = −0.672	*p* < 0.001 r = −0.602	*p* < 0.001 r = −0.379	*p* < 0.001 r = −0.783	*p* < 0.001 r = −0.786
Total hip BMD (g/sqcm)	*p* = 0.002 r = −0.405	*p* < 0.001 r = −0.754	*p* < 0.001 r = −0.726	*p* < 0.001 r = −0.432	*p* < 0.001 r = −0.775	*p* < 0.001 r = −0.823
**BT Group**						
Lumbar BMD (g/sqcm)	*p* = 0.417 r = −0.151	*p* = 0.112 r = −0.319	*p* = 0.100 r = −0.331	*p* = 0.066 r = −0.336	*p* = 0.069 r = −0.371	*p* = 0.096 r = −0.343
Femoral neck BMD (g/sqcm)	*p* = 0.013 r = −0.500	*p* < 0.001 r = −0.905	*p* < 0.001 r = −0.862	*p* = 0.016 r = −0.473	*p* < 0.001 r = −0.845	*p* < 0.001 r = −0.869
Total hip BMD (g/sqcm)	*p* = 0.299 r = −0.219	*p* = 0.009 r = −0.576	*p* = 0.029 r = −0.514	*p* = 0.226 r = −0.246	*p* = 0.006 r = −0.606	*p* = 0.019 r = −0.550

## Data Availability

The original contributions presented in this study are included in the article. Further inquiries can be directed to the corresponding author.

## Abbreviations

The following abbreviations were used in this manuscript:

ACTH	Adrenocorticotropic hormone
BMI	Body mass index
BMD	Bone mineral density
BTMs	Bone turnover markers
BT	Bilateral tumors
cs-1mg-DST	Second-day plasma cortisol after 1 mg dexamethasone testing
DXA	Dual-Energy X-ray Absorptiometry
DST	Dexamethasone suppression test
FRAX	Fracture risk estimator tool
HF	10-year fracture risk for hip fracture
HR-pQCT	High-resolution peripheral quantitative CT
IQR	Interquartile interval
MOF	10-year fracture risk for major osteoporotic
MMAs	Mineral metabolism assays
N	Number of patients
PTH	Parathyroid hormone
P1NP	Procollagen-N-terminal-peptide
Q	Quartile
SD	Standard deviation
TBS	Trabecular bone score
UT	Unilateral tumor
